# Self‐Assembled 2D VS_2_/Ti_3_C_2_T_x_ MXene Nanostructures with Ultrafast Kinetics for Superior Electrochemical Sodium‐Ion Storage

**DOI:** 10.1002/advs.202304465

**Published:** 2023-08-27

**Authors:** Pin Ma, Zehao Zhang, Jian Wang, Haibo Li, Hui Ying Yang, Yumeng Shi

**Affiliations:** ^1^ Ningxia Key Laboratory of Photovoltaic Materials School of Materials and New Energy Ningxia University Yinchuan 750021 China; ^2^ Pillar of Engineering Product Development Singapore University of Technology and Design 8 Somapah Road Singapore 487372 Singapore; ^3^ International Collaborative Laboratory of 2D Materials for Optoelectronics Science and Technology of Ministry of Education College of Optoelectronic Engineering Shenzhen University Shenzhen 518060 China

**Keywords:** MXene nanostructures, self‐assemble, sodium‐ion storage

## Abstract

Constructing nanostructures with high structural stability and ultrafast electrochemical reaction kinetics as anodes for sodium‐ion batteries (SIBs) is a big challenge. Herein, the robust 2D VS_2_/ Ti_3_C_2_T_x_ MXene nanostructures with the strong Ti─S covalent bond synthesized by a one‐pot self‐assembly approach are developed. The strong interfacial interaction renders the material of good structural durability and enhanced reaction kinetics. Meanwhile, the enlarged and few‐layered MXene nanosheets can be easily obtained according to this interaction, providing a conductive network for sufficient electrolyte penetration and rapid charge transfer. As predicted, the VS_2_/MXene nanostructures exhibit an extremely low sodium diffusion barrier confirmed by DFT calculations and small charge transfer impedance evidenced by electrochemical impedance spectroscopy (EIS) analysis. Therefore, the SIBs based on the VS_2_/MXene electrode present first‐class electrochemical performance with the ultrahigh average initial columbic efficiency of 95.08% and excellent sodium‐ion storage capacity of 424.6 mAh g^−1^ even at 10 A g^−1^. It also shows an outstanding sodium‐ion storage capacity of 514.2 mAh g^−1^ at 1 A g^−1^ with a capacity retention of nearly 100% within 500 times high‐rate cycling. Such impressive performance demonstrates the successful synthesis strategy and the great potential of interfacial interactions for high‐performance energy storage devices.

## Graphical abstract

In this study, the robust 2D VS_2_/ Ti_3_C_2_T_x_ MXene nanostructures with the strong Ti─S covalent bond synthesized by a one‐pot self‐assembly approach are developed. The strong interfacial interaction renders the material of good structural durability and enhanced reaction kinetics, leading to the first‐class electrochemical performance of sodium‐ion batteries based on the VS_2_/MXene electrode.

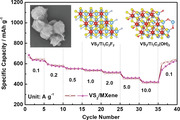

## 1 Introduction

Two‐dimensional (2D) nanostructures have been extensively adopted as anode materials for sodium‐ion batteries (SIBs) due to their unusual physical/chemical properties.^[^ ^1^, ^2^, ^3^, ^4^, ^5^ ^]^ Compared with zero‐dimensional (0D) and one‐dimensional (1D) nanostructures, 2D nanostructured electrodes show larger and more active spaces and locations, contributing to the rapid electrochemical reaction kinetics.^[^ ^6^, ^7^ ^]^ However, 2D materials are very easy to aggregate and self‐stack, which inevitably limits the sodium storage performance. Therefore, in the past decade, great efforts have been devoted to designing new 2D nanostructured electrodes for high‐performance SIBs.

Recently, transition metal carbides, nitrides, and carbonitrides (MXenes), as emerging star 2D materials have gained much attention from researchers.^[^ ^8^, ^9^, ^10^, ^11^, ^12^ ^]^ Although it shows high electronic conductivity, tunable interlayer spacing, and abundant surface groups, its application in SIBs still suffers from the tendency to stack and the low intrinsic capacity.^[^ ^13^, ^14^, ^15^, ^16^ ^]^ Moreover, it has been demonstrated that the ‐F and ‐OH terminal surface groups on MXenes could decrease the ionic conductivity, thus severely impeding the ion transport and restricting sodium storage.^[^ ^17^, ^18^ ^]^ Many strategies have been proposed to address the above‐mentioned challenges, such as synthesizing the single‐/few‐layer MXenes,^[^ ^19^, ^20^, ^21^, ^22^ ^]^ expanding the interlayer spacing,^[^ ^23^, ^24^, ^25^, ^26^ ^]^ and constructing the MXene‐based composite materials.^[^ ^27^, ^28^, ^29^, ^30^, ^31^, ^32^ ^]^

Among them, MXene‐based composite materials could not only prevent the agglomeration/accumulation of MXenes, but combine the advantages of single components while making up for the relevant shortcomings to achieve improved performances for SIBs. For instance, Meng et al.^[^ ^33^ ^]^ synthesized the composite of MXene nanosheets and black phosphorus quantum dots as the anode electrode for SIBs. The formation of P─O─Ti interfacial bonds accelerates the interfacial electron transfer, enabling fast and stable energy storage. Guo et al.^[^ ^34^ ^]^ reported the phosphorene/MXene nanoarchitecture and confirmed that the sodium diffusion kinetics in the phosphorene/Ti_3_C_2_F_2_ have been significantly enhanced. In particular, loading transition metal sulfides including layered sulfide materials (LTMD)^[^ ^35^, ^36^, ^37^, ^38^ ^]^ and nonlayered materials^[^ ^39^, ^40^, ^41^, ^42^, ^43^ ^]^ on MXenes is of great significance to enhance the performance of SIBs. Because of the ability to undergo conversion or/and alloying electrochemical mechanisms, the sulfide/MXene composites almost all show an improvement in specific capacities and stabilities.

Nevertheless, there are still a few important problems that need to be solved and understood. 1) The preparation for high‐quality few‐layered MXenes, especially in composites, is still a big challenge, which leads to insufficient exposure of the active site, resulting in the low concentration loading and poor utilization of active materials. 2) The weak interaction force between MXene and the second phase induces large electrochemical polarizations and slow interfacial charge transfer, limiting the high‐rate performance of SIBs. 3) The formation of an unstable solid electrolyte interface inevitably causes low initial columbic efficiencies (ICE) and rapid capacity degradation. 4) Among many studies of MXene‐based composites, few attempts have been made to understand the underlying mechanisms for sodium‐ion storage. Overall, there is still a long way to go for the widespread application of MXene‐based anodes for SIBs

As a representative member of LTMDs,^[^ ^44^, ^45^, ^46^, ^47^ ^]^ VS_2_ monolayer has the typical metallic feature and faster atom diffusion rate than graphite and MoS_2_.^[^ ^48^ ^]^ However, the limited specific surface area and volume variations of VS_2_ during charge/discharge processes lead to poor cycling stability and low reversible capacity of batteries.^[^ ^49^, ^50^ ^]^ Lately, theoretical investigations have found that the integration of VS_2_ with MXenes could greatly enhance the electronic conductivity and adsorption strength, which is proposed to be a preferable anode material for SIBs.^[^ ^51^ ^]^ Therefore, it is very exciting yet highly challenging to fabricate the unique VS_2_/MXene heterostructure, then significantly accelerate the kinetics and achieve high‐performance SIBs. More recently, VS_2_/MXene hybrid was successfully produced by a conventional hydrothermal synthesis approach and applied in supercapacitors^[^ ^52^ ^]^ and Li‐S batteries.^[^ ^53^ ^]^ It is noticeable that the peculiar structure has a great impact on the electrochemical energy storage performance. Inspired by these results, considering the high requirements of SIB electrode materials, it is necessary to effectively optimize the composition and structure of VS_2_/MXene composites to obtain superior electrochemical sodium‐ion storage.

Hence, in this work, the 2D VS_2_/MXene nanostructures are elaborately and successfully synthesized through the self‐assembly strategy of well‐dispersed few‐layer MXene nanosheets on VS_2_ without any surfactants followed by a freeze‐drying process and used as the anodes for SIBs for the first time. The enlarged, few‐layer MXenes can play the role of a 3D connected, conductive network and buffer matrix for VS_2_ to fully deliver its high capacity advantage. More importantly, it is found that there is the formation of strong Ti─S covalent bonds between two components, which is favorable to the efficient interfacial electron transfer and improves the surface capacitive contribution for sodium storage. In addition, according to the DFT calculations, the sodium absorption energy and diffusion energy barrier for the interface have been significantly decreased, enabling ultrafast kinetics for electrochemical reactions. Consequently, the 2D VS_2_/MXene nanostructures electrode exhibits an ultrahigh ICE (95.08%), impressive rate capacity of 424.6 mAh g^−1^ at 10 A g^−1^, and outstanding cycling stability of 514.2 mAh g^−1^ with capacity retention nearly 100% within 500 cycles at 1 A g^−1^. These results highlight the role of 2D nanostructured electrodes for high‐performance rechargeable batteries and demonstrate the importance of interface interaction in effectively boosting the kinetics and stability of electrodes.

## 2 Results and Discussion

**Figure 1:**
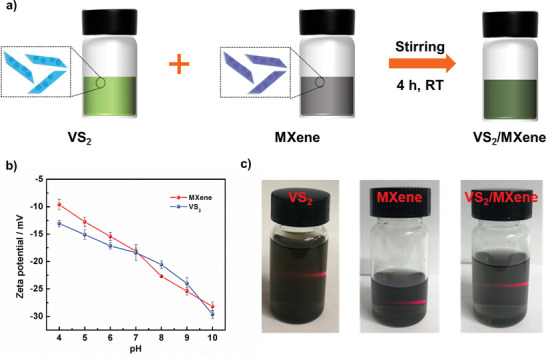
a) Schematic drawing for the fabrication of the VS_2_/MXene nanostructure through a simple self‐assembly method. b) Zeta potential properties for VS_2_ and MXene materials. c) Digital photographs of the VS_2_, MXene, and VS_2_/MXene mixture colloids.

The overall fabrication process of the VS_2_/MXene nanostructure is described in **Figure** **1**a, which involves the separate synthesis of VS_2_ and MXene, followed by the self‐assembly of MXene on VS_2_ nanosheets. On one hand, the VS_2_ nanosheets are achieved through the hydrothermal method with later ultrasonic treatment in DI water. On the other hand, MXene nanosheets are synthesized by selectively etching the pristine Ti_3_AlC_2_ in LiF‐HCl concentrated solution and subsequent manual‐shaking approach. Finally, the VS_2_/MXene nanostructures are fabricated by assembling MXene onto VS_2_ nanosheets (weight ratio is 1:5) driven by Van Der Waals forces with the assistance of longtime stirring. Zeta potential properties of nanosheets are first measured and shown in Figure 1b. It can be seen that the surfaces of both VS_2_ and MXene are negatively charged in the pH range of 4–10. Interestingly, at a pH of 7, the values of zeta potential of VS_2_ and MXene are almost the same −18.37 and −18.10 mV, respectively. Noted that the pH for the solution used in the synthesis for hybrid materials is very close to 7, which is more favorable to the interaction between two components. Figure 1c presents the optical images for the dispersions of VS_2_ and MXene. The strong Tyndall scattering effect can be clearly observed when directing a side laser light on the colloids, demonstrating the good and stable dispersion of materials in aqueous media. Noteworthy, the mixture of two colloids of VS_2_ and MXene shows a homogeneous dispersion with no apparent precipitation due to the electrostatic repulsion, indicating the good compatibility between the two types of nanosheets. Moreover, the zeta potential of VS_2_/MXene is measured as −20.3 mV, which means that the hybridization does not change the overall state of the surface charge.

**Figure 2:**
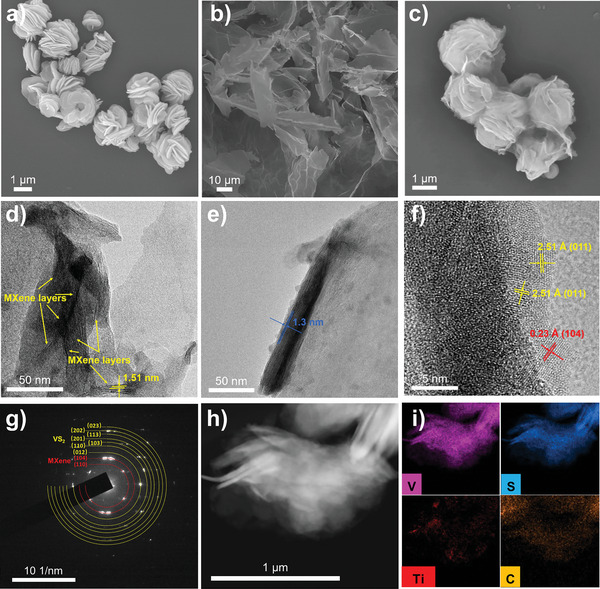
a–c) SEM images of VS_2_, MXene, and VS_2_/MXene nanostructures. d,e) TEM images of VS_2_/MXene and MXene. f,g) HRTEM image and the SAED pattern for VS_2_/MXene hybrid. h,i) STEM image of VS_2_/MXene hybrid and the corresponding EDS mapping of V, S, Ti, and C element distribution.

The morphologies and microstructures of materials are measured by SEM and TEM. As shown in **Figure** **2**a, the monodispersed and homogeneous VS_2_ microflowers are formed from a large number of nanosheets with a thickness of ≈20–50 nm and a diameter of ≈1.5 µm. Besides, the SEM image for MXene (Figure 2b) clearly describes a self‐stacked and crinkled sheet morphology with an ultrathin thickness. After the self‐assembly process, the VS_2_ nanosheets are tightly wrapped by MXene as presented in Figure 2c and Figure S1 and S2 (Supporting Information). These tightly packed MXene nanosheets could not only improve the electronic conductivity of VS_2_, but also act as a matrix to cushion the volume expansion of VS_2_ during the charge/discharge process. Meanwhile, the few‐layer characteristic of MXene can be observed by TEM (Figure 2d) with a larger layer spacing of 1.51 nm than that of pure MXene (1.3 nm, Figure 2e). Moreover, the HRTEM image for VS_2_/MXene (Figure 2f) displays clear lattice fringes with d‐spacing of 2.51 and 2.30 Å, corresponding to the (011) plane of VS_2_ (JCPDS No. 89–1640) and (104) plane of MXene,^[^ ^54^, ^55^ ^]^ respectively. The SAED pattern (Figure 2g) reveals the in‐plane diffraction of planes for both VS_2_ and MXene, further proving the existence of both nanosheets. In addition, the STEM image (Figure 2h) and corresponding EDS spectrum (Figure 2i) shows the distribution of V, S, Ti, and C, displaying the successful combination of VS_2_ and MXene through the self‐assembly process.

**Figure 3:**
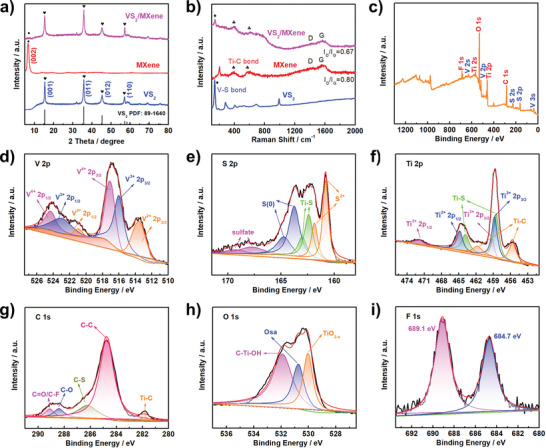
a,b) XRD patterns and Raman spectra of VS_2_/MXene, VS_2_, and MXene. c) The full survey XPS spectrum of VS_2_/MXene and the corresponding high‐resolution spectra in the d–i) V 2p, S 2p, Ti 2p, C 1s, O1s, and F 1s region.

To identify the crystal structure of VS_2_/MXene nanostructures, XRD patterns are employed. As shown in **Figure** **3**a, the strong diffraction peaks for VS_2_/MXene are well matched with the pristine VS_2_ (JCPDS No. 89–1640), and a weak peak below 10° is attributed to the MXene, which may be due to either the small amount or the low crystallinity of MXene in the composite. Figure 3b displays the Raman spectra of three samples. Despite the weakened peak intensities, the peaks for VS_2_ and MXene signals are well identified in the hybrid. Meanwhile, in comparison with pristine MXene, the ratio for the D band and G band of VS_2_/MXene decreases from 0.80 to 0.67, suggesting a higher graphitization degree. Besides, the XPS spectrum is utilized to visualize the surface chemical compositions of materials. Figure 3c shows the survey XPS spectrum of VS_2_/MXene nanostructures, where the characteristic peaks of V, S, Ti, C, O, and F can be clearly seen in the chart. The high‐resolution V 2p spectrum of VS_2_/MXene (Figure 3d) can be deconvoluted into three spin‐orbit doublets at 524.3/517.1, 523.0/516.0, and 521.0/513.6 eV, which are due to the V4^+^, V3^+^, and V^2+^, respectively, and consistent with the XPS spectrum for pristine VS_2_ shown in Figure S3a (Supporting Information).^[^ ^53^, ^56^, ^57^ ^]^ The core high‐resolution S 2p XPS spectrum of VS_2_/MXene (Figure 3e) displays four peaks of S 2p_1/2_ and S 2p_3/2_ at 169.3/167.7, 164.7/163.8, 163.0/162.4, and 161.9/160.8 eV, which are attributed to sulfate, S(0), Ti─S bond and S^2+^, respectively.^[^ ^42^, ^58^, ^59^ ^]^ Compared with the S 2p spectra of pristine VS_2_ (Figure S3b, Supporting Information), the formation of a strong covalent Ti─S chemical bond is favorable to the stability and charge transfer of materials. The deconvolution of the Ti 2p spectrum for VS_2_/MXene (Figure 3f) reveals four pairs of peaks located at 471.9/459.2, 463.8/458.5, 464.9/458.8, and 461.8/455.6 eV, corresponding to Ti^3+^, Ti^2+^, Ti─S bond, and Ti─C bond, respectively.^[^ ^23^, ^26^, ^60^ ^]^ In comparison with Ti 2p spectra of pristine MXene (Figure S4, Supporting Information), this result further confirms the existence of the Ti─S bond at the interfaces between VS_2_ and MXene. The detailed charge transfer between Ti and S will be discussed in the following simulation section. The C 1s spectrum of VS_2_/MXene (Figure 3g) exhibits five peaks at 289.1, 288.4, 286.2, 284.7, and 281.8 eV, which can be assigned to C═O/C═F, C─O, C─S, C─C, and Ti─C, respectively.^[^ ^23^, ^34^, ^59^ ^]^ The O 1s XPS in VS_2_/MXene (Figure 3h) appears at three different peaks located at 531.9, 530.7, and 530.0 eV, representing the surface C─Ti─OH species, surface active O (Osa), and TiO_2‐x_ species, respectively.^[^ ^29^, ^61^, ^62^ ^]^ The high‐resolution F 1s spectrum (Figure 3i) can be deconvoluted into two peaks at 689.1 and 684.7 eV, which are ascribed to the surface metal‐fluorine binding.^[^ ^62^, ^63^, ^64^ ^]^ In addition, Figure S5a (Supporting Information) presents the N_2_ adsorption–desorption isotherms of three samples. It can be observed that the BET surface area for VS_2_/MXene is 55.0 m^2^ g^−1^, which is much higher than that of pure VS_2_ (12.8 m^2^ g^−1^) and slightly lower than that of pure MXene (57.2 m^2^ g^−1^). The slight decrease relative to pure MXene is attributed to the successful loading of VS_2_ and the large increase compared with pure VS_2_ demonstrates the great advantages of few‐layer MXenes. Furthermore, the pore size distribution curves (Figure S5b, Supporting Information) indicate the rich micropores/mesopores with an average pore size of 15 nm in VS_2_/MXene nanostructures. The high surface area and porous structure of VS_2_/MXene are expected to expose more active sites for sodium storage as well as make fully close contact between the electrode and electrolyte, which is beneficial for the fast diffusion of sodium ions.

**Figure 4:**
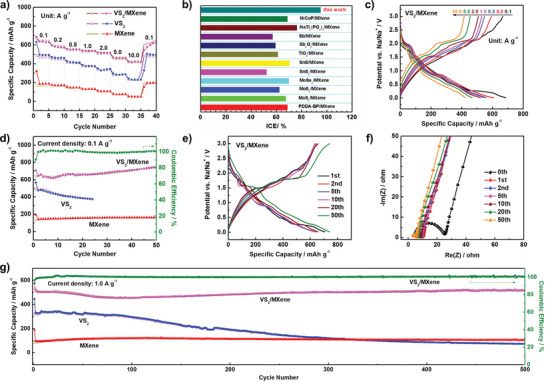
a) The rate capability of VS_2_/MXene electrode at various current densities from 0.1 to 10.0 A g^−1^. b) ICE comparison of typical MXene‐based materials in SIBs. c) The galvanostatic charge–discharge curves of VS_2_/MXene electrode at various current densities from 0.1 to 10.0 A g^−1^. d) Cycling performance of various electrodes at 0.1 A g^−1^. e) The galvanostatic charge–discharge curves of VS_2_/MXene electrode at a current density of 0.1 A g^−1^. f) Nyquist plots of the VS_2_/MXene electrode before cycling and after different cycles at full charge states. g) Long‐term cycling performance of various electrodes at 1.0 A g^−1^.

The sodium storage capacities of all samples are evaluated by assembling them into the 2032 coin‐type half cells with sodium metal as the counter electrodes. **Figure** **4**a reveals the rate performance at current densities ranging from 0.1 to 10.0 A g^−1^. Compared with the original VS_2_ and MXene, the VS_2_/MXene nanostructures exhibit the highest specific capacities. The initial discharge and charge capacities for VS_2_/MXene electrodes at 0.1 A g^−1^ are 685.5 and 667.1 mAh g^−1^ with a high coulombic efficiency of 96.8%. The ICE of VS_2_ and MXene are only calculated as 77.7% and 53.3%, respectively. Meanwhile, the five different batteries with VS_2_/MXene anode deliver an ultrahigh average ICE value of 95.08% (Figure S6, Supporting Information), which is greatly higher than that of the previously reported MXene‐based materials (Figure 4b; Table S1, Supporting Information) for SIBs. Impressively, at the current density of 10.0 A g^−1^, the VS_2_/MXene electrode still shows an outstanding capacity of ≈424.6 mAh g^−1^, almost more than twice that of VS_2_ (241.1 mAh g^−1^) and 8 times of individual MXenes (53.6 mAh g^−1^). This result suggests the excellent rate performance for VS_2_/MXene nanostructures, which could be further verified by a capacity of 628 mAh g^−1^ as the current density goes back to 0.1 A g^−1^. Besides, the shape of corresponding galvanostatic charge–discharge curves (Figure 4c; Figure S7, Supporting Information) does not change much except for the gradual decrease of the capacities together with the improvement of the current densities. Furthermore, VS_2_/MXene nanostructures also present excellent cycling stability. Figure 4d compares the cycling performances of three samples at a current density of 0.1 A g^−1^, and the corresponding charge/discharge profiles are shown in Figure 4e and Figure S8 (Supporting Information). It can be observed that the capacity of VS_2_/MXene electrode decreases first and then increases, possessing an obvious uplifting phenomenon along with cycling. This is mainly due to the pillar effect between VS_2_ and MXenes, referring to the consequence of the volume expansion of materials during subsequent cycling.^[^ ^59^, ^65^ ^]^ As a result, the VS_2_/MXene electrode delivers a capacity of 742.4 mAh g^−1^ after 50 cycles, which is even higher than the first discharge capacity of 706.7 mAh g^−1^. By contrast, the individual VS_2_ electrode undergoes a serious capacity degradation, while MXene electrode shows high reversibility. Figure 4f presents the Nyquist plots of the electrodes before and after selected cycles. All plots have one semicircle in the high frequencies and a straight line in the low frequencies, which can be ascribed to the charge transfer impedance of the electrolyte‐electrode interface and the Warburg impedance, respectively. We can see that the charge transfer impedance dramatically decreases after cycling and keeps almost constant upon long‐term cycling, suggesting the good stability of the electrode. This is mainly ascribed to the formation of the SEI layer during the initial cycles and the better electrochemical contact between electrolyte and electrode after cycling.^[^ ^66^, ^67^, ^68^ ^]^ Meanwhile, the VS_2_/MXene electrode shows the smallest charge transfer impedance compared with that of individual components (Figure S9, Supporting Information), indicating the enhanced sodium ion diffusion efficiency. Moreover, Figure 4g shows the long‐term cycling performance of electrodes for 500 cycles at a current density of 1.0 A g^−1^. It still can be seen that the capacity of VS_2_/MXene electrode decreases initially and then increases. Consequently, after 500 cycles, the capacity retention of VS_2_/MXene is almost 100%, indicating the VS_2_/MXene nanostructures possess the high‐efficient and stable sodium ions storage mode.

**Figure 5:**
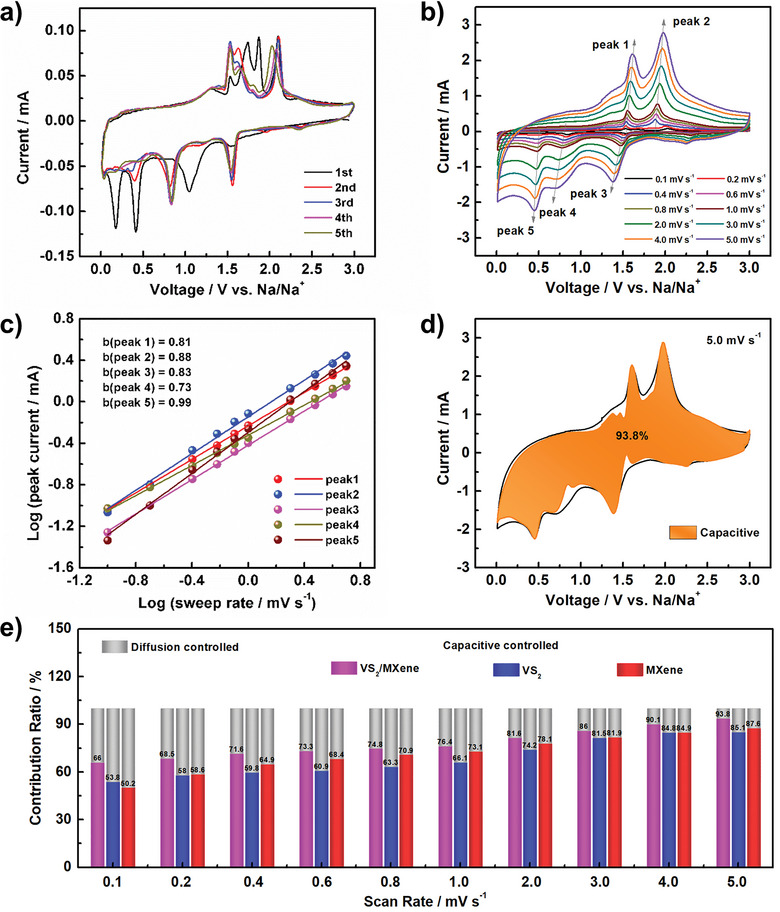
a,b) CV curves of VS_2_/MXene electrode at 0.1 mV s^−1^ and different scan rates. c) Relationship between log (peak current) versus log (sweep rate). d) Capacitive‐controlled and diffusion‐controlled contributions for VS_2_/MXene at 5.0 mV s^−1^. e) Normalized contribution ratio of capacitive‐controlled capacities for three electrodes at different scan rates.

To understand the charge transfer kinetics and explain the superior sodium ion storage performance of VS_2_/MXene anode, the initial five cyclic voltammograms (CV) curves are first investigated with a voltage window of 0.1–3.0 V at 0.1 mV s^−1^, and the results are shown in **Figure** **5**a. In the first cathodic scan, two peaks at 0.42 and 0.18 V are detected and gradually disappear in the following sweeps, belonging to the irreversible reaction related to the SEI formation and resulting in the irreversible capacity.^[^ ^69^ ^]^ Another two peaks at 1.57 and 1.05 V are ascribed to the multi‐step sodium‐ion insertion of VS_2_ and MXene, which then shift to the lower potential of 1.56 and 0.82 V in the following cycles, respectively.^[^ ^28^, ^70^ ^]^ During the anodic scan, peaks between 1.3 and 2.3 V can be observed, corresponding to the sodium‐ion extraction out of the materials. The well‐overlapped curves for 2–5 cycles demonstrate the satisfied electrochemical reversibility of the VS_2_/MXene nanostructures during charge/discharge processes. Furthermore, the CV curves at different scan rates from 0.1 to 5.0 mV s^−1^ for VS_2_/MXene are recorded and depicted in Figure 5b. There is a small peak shift with the increase of the scan rate, which is attributed to the low polarization of VS_2_/MXene in the dimethylether (DME) electrolyte. Besides, a related analysis can be conducted according to the relationship between the measured peak current (*i*) and scan rate (*v*) described by the following equation

(1) $$i=a\cdot v^{b}$$

where *a* and *b* are both adjustable values. Therefore, the slope of the corresponding log(v)‐log(i) plots (Figure 5c) determines the value of *b*. When the value of *b* is close to 1 or 0.5, the system is mainly dominated by surface capacitive or diffusion processes, respectively. As can be seen from Figure 5c, the values of *b* for all peaks approach 1, indicating the battery is mainly controlled by the surface capacitive behavior. Meanwhile, the specific contribution from diffusion‐controlled and surface capacitive‐controlled at a fixed scan rate can be calculated according to the equation:

(2) $$i\left(V\right)=k_{1}v+k_{2}v^{1/2}$$

where i(V) represents the current at a given voltage, and *k*_1_v and *k*_2_v^1/2^ are the surface capacitive‐controlled and diffusion‐controlled contributions, respectively. As shown in Figure 5d, the surface capacitive‐controlled contribution for VS_2_/MXene electrode reaches 93.8% of the total capacity at a scan rate of 5.0 mV s^−1^. In addition, the CV curves for individual VS_2_ and MXene are also detected and the relevant pictures are given in Figure S10 (Supporting Information). Moreover, as shown in Figure 5e, the VS_2_/MXene nanostructures always deliver a larger capacitive component than the individual VS_2_ and MXene electrodes at different scan rates. It is believed that this high surface capacitive‐dominated capacity contributes to the fast electrochemical reactions and the high rate performance of VS_2_/MXene electrodes.

**Figure 6:**
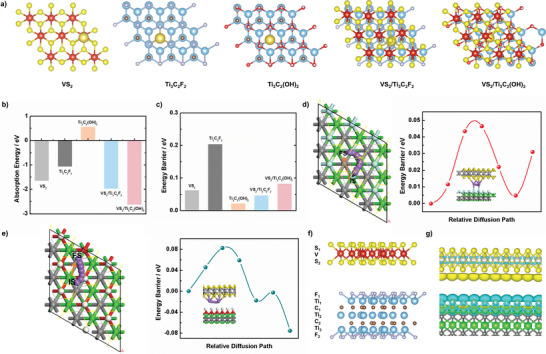
a) Top views of Na adsorption sites on the surface, b) the calculated binding energies of the most stable Na adsorption sites, and c) the energy barrier of sodium diffusion for VS_2_, Ti_3_C_2_F_2_, Ti_3_C_2_(OH)_2_, VS_2_/Ti_3_C_2_F_2_, and VS_2_/Ti_3_C_2_(OH)_2_. d,e) Diffusion path of one sodium atom diffusion on the interface of VS_2_/Ti_3_C_2_F_2_ and VS_2_/Ti_3_C_2_(OH)_2_. f,g) Side view and the graphical display of charge differences for VS_2_/Ti_3_C_2_F_2_ structure.

The DFT calculations are further performed to investigate the absorption and diffusion characteristics of sodium in the VS_2_/MXene nanostructures, which could be accountable for the possible mechanisms of superior sodium storage performance. The VS_2_/MXene heterostructures are obtained by a monolayer of VS_2_ and Ti_3_C_2_F_2_/Ti_3_C_2_(OH)_2_ stacked in the vertical direction through the interaction of Van De Waals forces.^[^ ^71^ ^]^ The monolayer VS_2_, Ti_3_C_2_F_2_/Ti_3_C_2_(OH)_2,_ and their heterostructures are all investigated. **Figure** **6**a presents the most stable adsorption site for sodium absorption in the above models and the corresponding absorption energies for a sodium atom are shown in Figure 6b. It can be seen that the VS_2_/Ti_3_C_2_F_2_ and VS_2_/Ti_3_C_2_(OH)_2_ present a very high absorption energy toward sodium atoms, which is much better than that of the individual components. This confirms that the heterostructure is beneficial for sodium storage. Meanwhile, the energy barrier for sodium diffusion is also investigated and shown in Figure 6c‐e and Figure S11 (Supporting Information). Noted that the energy barrier of one sodium atom diffusing along with the VS_2_/Ti_3_C_2_F_2_ interface is lower than that of diffusing along the surface of monolayer VS_2_ and Ti_3_C_2_F_2_. The difference between the interface and Ti_3_C_3_F_2_ is 0.157 eV (DFT accuracy is around 0.1 eV), so it can be considered that the formation of the interface enhances the sodium diffusion kinetics. Moreover, the energy barrier of sodium diffusion in VS_2_/Ti_3_C_2_F_2_ (0.0464 eV) is much lower than the previously reported sodium diffusion barrier in other 2D heterostructures, such as BP/Ti_3_C_2_F_2_ (0.1729 eV)^[^ ^34^ ^]^ and MoSe_2_/MXene (0.095 eV).^[^ ^55^ ^]^ This low energy barrier ensures fast ions movement during the sodiation/desodiation process, leading to the high rate performance for SIBs. Besides, the energy barrier for Ti_3_C_2_(OH)_2_ is calculated as 0.0216 eV, whereas the barrier for the interface of VS_2_/Ti_3_C_2_(OH)_2_ is 0.0823 eV. Due to the accuracy of the DFT, the difference of only 0.06 eV can be considered that there is little difference in the diffusion of sodium atoms between the two structures and the sodium spreads out very quickly in both. Furthermore, the galvanostatic intermittent titration technique (GITT) profiles shown in Figure S12 (Supporting Information) demonstrate the lower overpotential for VS_2_/MXene during the whole sodium storage reactions compared with the individual components, further verifying the fast diffusion of sodium.^[^ ^72^, ^73^, ^74^, ^75^ ^]^ Next, the electron excursion is determined by the calculation of Bader charges. The side view for VS_2_/Ti_3_C_2_F_2_ structure is shown in Figure 6f and the corresponding calculated data is listed in Table S2 (Supporting Information). Besides, the graphical display of charge differences is also shown in Figure 6g to clearly demonstrate the charges in the interface. Compared with the pristine VS_2_ and Ti_3_C_2_F_2_, the interface shows the electron‐rich state of V and Ti, as well as the electron‐lack state of S, C, and F. Consequently, the charges for S ions, which could transfer to the Ti ions through Ti─S bond, further confirming the strong interaction between VS_2_ and MXene. Meanwhile, the charges for C atoms could transfer to V ions through F ions. Therefore, the interaction between VS_2_ and Ti_3_C_2_F_2_ can be depicted by the following reaction (the superscript values delegate the charge changes of the corresponding elements).

(3) $${\mathrm{VS}}_{2}+{\mathrm{Ti}}_{3}C_{2}F_{2}\to {\left[V\right]}^{-2.00}{\left[S\right]}^{+1.00}+{\left[{\mathrm{Ti}}_{3}\right]}^{-2.38}{\left[C_{2}\right]}^{+2.70}{\left[F_{2}\right]}^{+0.86}$$

Additionally, the simulation for VS_2_/Ti_3_C_2_(OH)_2_ structure is also performed and the results are shown in Figure S13 and Table S3 (Supporting Information). The charge transfer is very similar to that of VS_2_/Ti_3_C_2_F_2_ and the interaction between VS_2_ and Ti_3_C_2_(OH)_2_ can be given as follows.

(4) $$\begin{array}{c} {\mathrm{VS}}_{2}+{\mathrm{Ti}}_{3}C_{2}{\left(\mathrm{OH}\right)}_{2}\to {\left[V\right]}^{-2.00}{\left[S\right]}^{+1.05}+{\left[{\mathrm{Ti}}_{3}\right]}^{-2.38}{\left[C_{2}\right]}^{+2.68}\left({\left[O_{2}\right]}^{+1.40}\left[H_{2}\right]\right) \end{array}$$

## 3 Conclusion

In summary, a unique 2D VS_2_/MXene nanostructure is synthesized through the simple self‐assembly strategy to prevent the agglomeration/accumulation of MXenes and protect VS_2_ to fully deliver its high capacity advantage. The enlarged, few‐layer MXene nanosheets are tightly anchored into the VS_2_ nanosheets with the strong interface interaction as well as the formation of Ti─S covalent bond, endowing the material with high structural durability and accelerating the electrochemical activity. Besides, the VS_2_/MXene anode for SIBs possesses a low sodium diffusion barrier (0.0464 eV) calculated by DFT, small charge transfer impedance, and high surface capacitive‐dominated contribution, demonstrating the ultrafast kinetics of electrode during the charge‐discharge processes. As a result, the fabricated SIBs exhibit ultrahigh average ICE, excellent rate capability, and prolonged cycling stability. The successful synthesis strategy would enable the VS_2_ for prospective anode materials for SIBs and can be extended to other novel nanostructures for impressive electrochemical performance.

## Conflict of Interest

The authors declare no conflict of interest.

## Supporting information

Supporting Information

## Data Availability

The data that support the findings of this study are available from the corresponding author upon reasonable request.
