# Supplementary material for: Self‐Assembled 2D VS2/Ti3C2Tx MXene Nanostructures with Ultrafast Kinetics for Superior Electrochemical Sodium‐Ion Storage
Source: Adv Sci (Weinh). 2023 Aug 27;10(31):2304465. doi: 10.1002/advs.202304465 (PMC10625112; doi:10.1002/advs.202304465)
Supplement: Supplementary file 1 — Supporting Information [file ADVS-10-2304465-s001.pdf]

ADVANCED SCIENCE
Open Access

Supporting Information

for *Adv. Sci.*, DOI 10.1002/advs.202304465

Self-Assembled 2D $VS_2/Ti_3C_2T_x$ MXene Nanostructures with Ultrafast Kinetics for Superior Electrochemical Sodium-Ion Storage

Pin Ma, Zehao Zhang, Jian Wang, Haibo Li*, Hui Ying Yang* and Yumeng Shi**

Supporting Information

Self-Assembled Two-Dimensional $VS_2/Ti_3C_2T_x$ MXene Nanostructures with Ultrafast Kinetics for Superior Electrochemical Sodium-Ion Storage

Pin Ma[1], Zehao Zhang[1], Jian Wang[1], Haibo Li[1,*], Hui Ying Yang[2,*], Yumeng Shi[3,*]

[1]Ningxia Key Laboratory of Photovoltaic Materials, School of Materials and New Energy, Ningxia University, Yinchuan, 750021, China

[2]Pillar of Engineering Product Development, Singapore University of Technology and Design, 8 Somapah Road, 487372, Singapore

[3]International Collaborative Laboratory of 2D Materials for Optoelectronics Science and Technology of Ministry of Education, College of Optoelectronic Engineering, Shenzhen University, Shenzhen 518060, China

Experimental details.

1. Materials synthesis

(1) *Preparation of MXene*

Typically, 1 g MAX (Ti_3AlC_2, 400 mesh) was added stepwise into 37.5 mL 9 M HCl and also contained 2.4 g LiF, stirring for half an hour at room temperature. The addition time is controlled at about half an hour in case the temperature is too high to cause oxidation. The etching process was then continued to be stirred for 24 h at 35 ^{o}C and washed thoroughly with deionized water for several times (4500 rpm, 5 min) until the upper layer of solution appeared the color of dark green. After that, 20 mL deionized water was added into the centrifugal tube followed by manually shaking for 1-2 h and centrifuging at 4500 rpm for 20 min with additional 20 mL deionized water. Finally, the upper solution was collected and stored in the refrigerator. 1 mL MXene solution was taken out and freeze-dried to measure its concentration.

(2) *Preparation of VS_2 nanosheets*

The preparation of VS_2 nanosheets is according to the literature[1]. Typically, 1 g PVP K-30 was first dissolved in the mixture of 30 mL deionized water and 2 mL ammonium hydroxide. Then 0.234 g

NH_4VO_3 was dissolved in sequence with continuous stirring. Then 1.5026 g C_2H_5NS (TAA) was added. The solution kept stirring 1 h at room temperature. After that, the solution was loaded into a Teflon-lined sealed autoclave and maintained at 180 °C for 20 h. The obtained suspension was centrifuged and the product was washed with deionized water and ethanol several times and then dried at 60 °C in a vacuum oven for overnight. Finally, the products were annealed at 300 °C for 2 h to obtain VS_2 naosheets.

(3) *Synthesis of VS_2/MXene*

40 mg VS_2 was firstly dispersed in the 40 mL deionized water to get a homogenous solution A. 8 mL MXene aqueous solution with concentration of 1 mg mL^{-1} was denoted as solution B. Solution B was then slowly added into solution A followed by stirrring for 4 h. The resultant dispersion was centrifuged for 10 min at 11000 rpm. Finally, the product was collected after freeze-drying.

2. Characterization

The morphology of the prepared materials was observed using a scanning electron microscope (SEM, Hitachi S-4800) together with associated energy-dispersive X-ray spectroscopy (EDX) and a transmission electron microscopy (TEM, JEOL JEM-2100F). X-ray diffraction (XRD) measurements were obtained by using a Bruker D8 Advance X-ray diffractometer with a Cu Kα radiation source over a 2θ range of 5-80°. The Raman spectra was perfomed with a confocal Raman system (WITec, α300R). An X-ray photoelectron spectroscopy (XPS, PHI Quantera II) with Al Kα source was used to analyze the surface electronic states of the powders. Nitrogen adsorption-desorption isotherms were carried out on a Quantachrome Autosorb IQ analyzer.

3. Electrochemical measurements

For making the anode electrodes, the slurry was prepared by mixing the samples (70 wt%), Ketjen black (20 wt%) and polyvinylidene fluoride (PVDF, 10 wt%) in 1-methyl-2-pyrrolidinone (NMP). Then the slurris were casted onto the Cu foils and dried in the vacuum oven at 70 °C for overnight. The assembled CR-2032 type coin cells were used the anode electrodes as the working electrodes and sodium foil as the counter and reference electrodes. The electrolyte was 1 M $NaPF_6$ dissolved into DME solution (DuoDuo-NP-035). Glass fibres (Advantec) were used as separators. The galvanostatic charge/discharge measurements, electrochemical impedance spectroscopic (EIS) and cyclic voltammetry (CV) were performed on the multichannel battery measurement system (Neware) and electrochemical workstation (IM6e) in the voltage of 0.01-3 V.

4. DFT calculations

All first-principles calculations were performed using the density functional theory (DFT) based on the Vienna ab initio simulation package (VASP) and projector augmented wave (PAW) method. The generalized gradient approximation (GGA) with the scheme of Perdew-Burke-Ernzerhof (PBE) was considered for the correlation energy and electron exchange. The supercells were set as Monkhorst-Pack 3×3×1 k mesh for the Brillouin zone sampling. The climbing image nudged elastic band (NEB) method was utilized to evaluate the diffusion/migration energy barrier. The Bader charge analysis was performed using the Bader Charge Analysis Code.

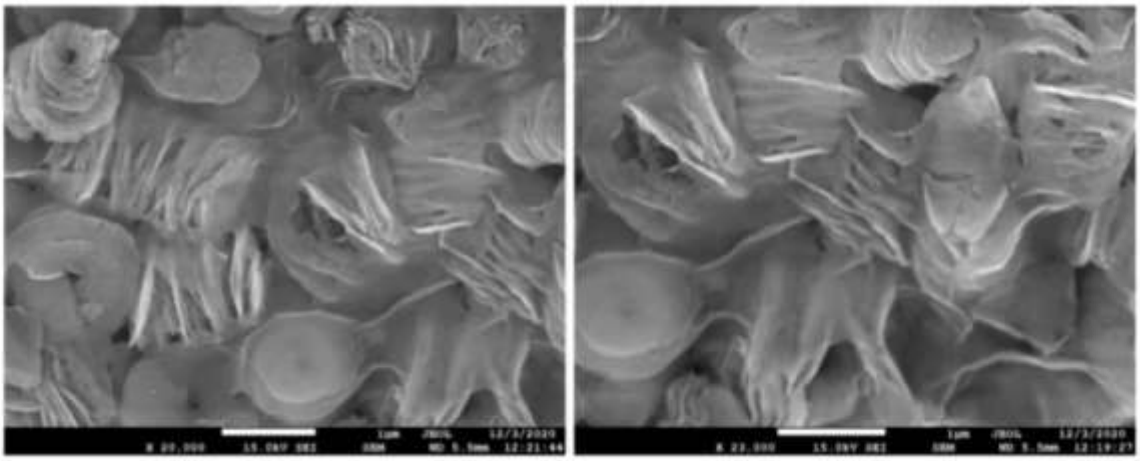

Figure S1. SEM images of VS_2/MXene nanostructures.

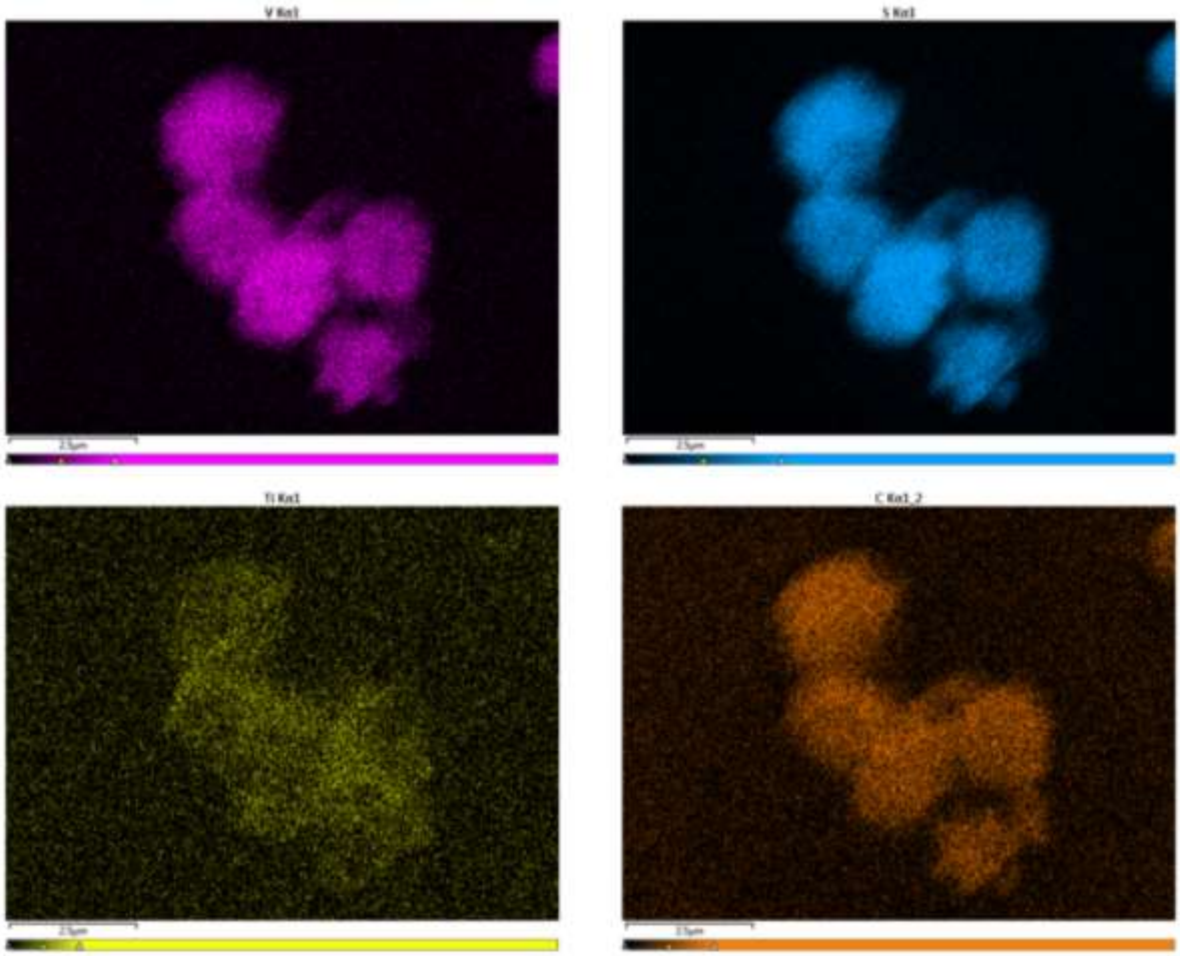

Figure S2. The corresponding EDS images of VS_2/MXene nanostructures shown in Figure 2c.

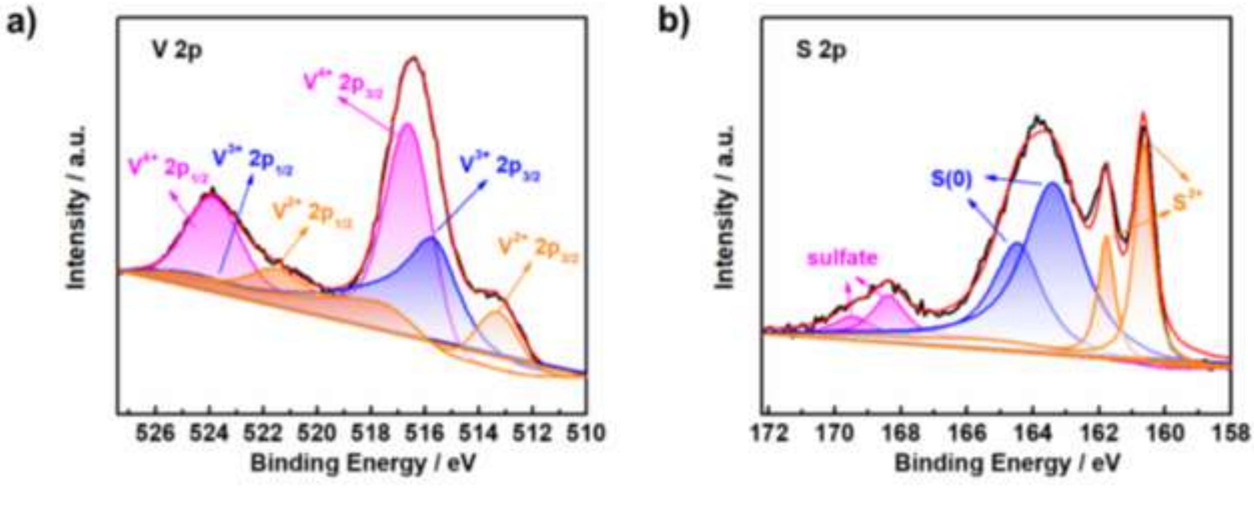

Figure S3. High-resolution V 2p and S 2p spectrum of pristine VS_2.

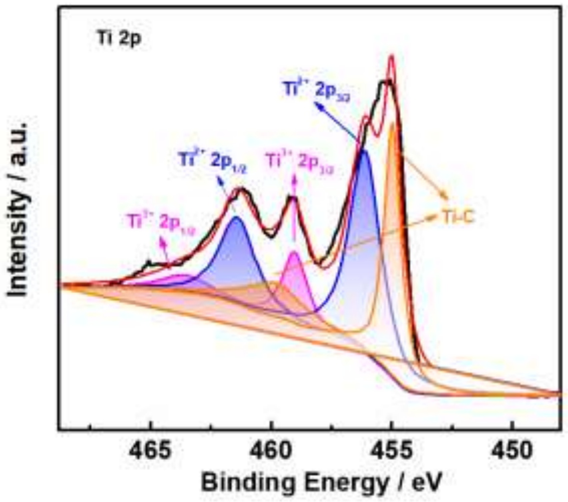

Figure S4. High-resolution Ti 2p spectrum of pristine MXene.

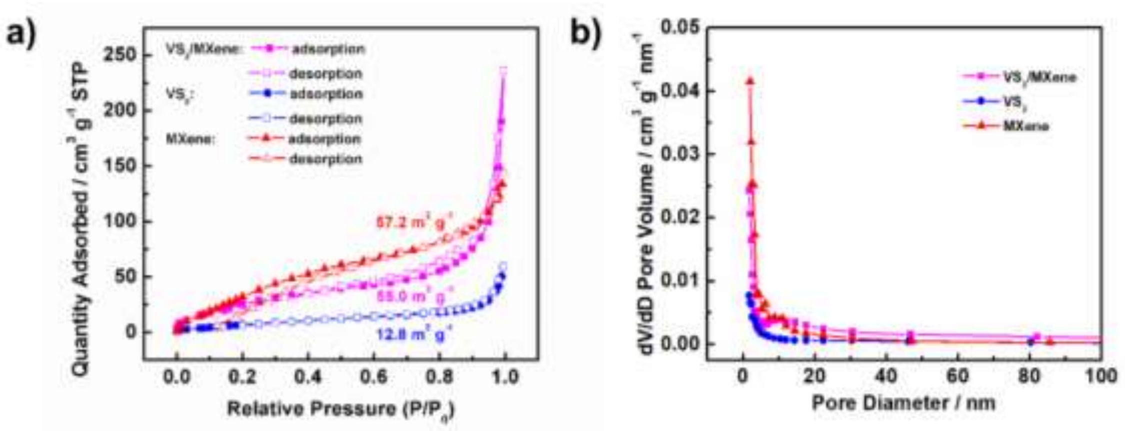

Figure S5. (a) Nitrogen adsorption-desorption isotherms and (b) pore size distribution of three samples.

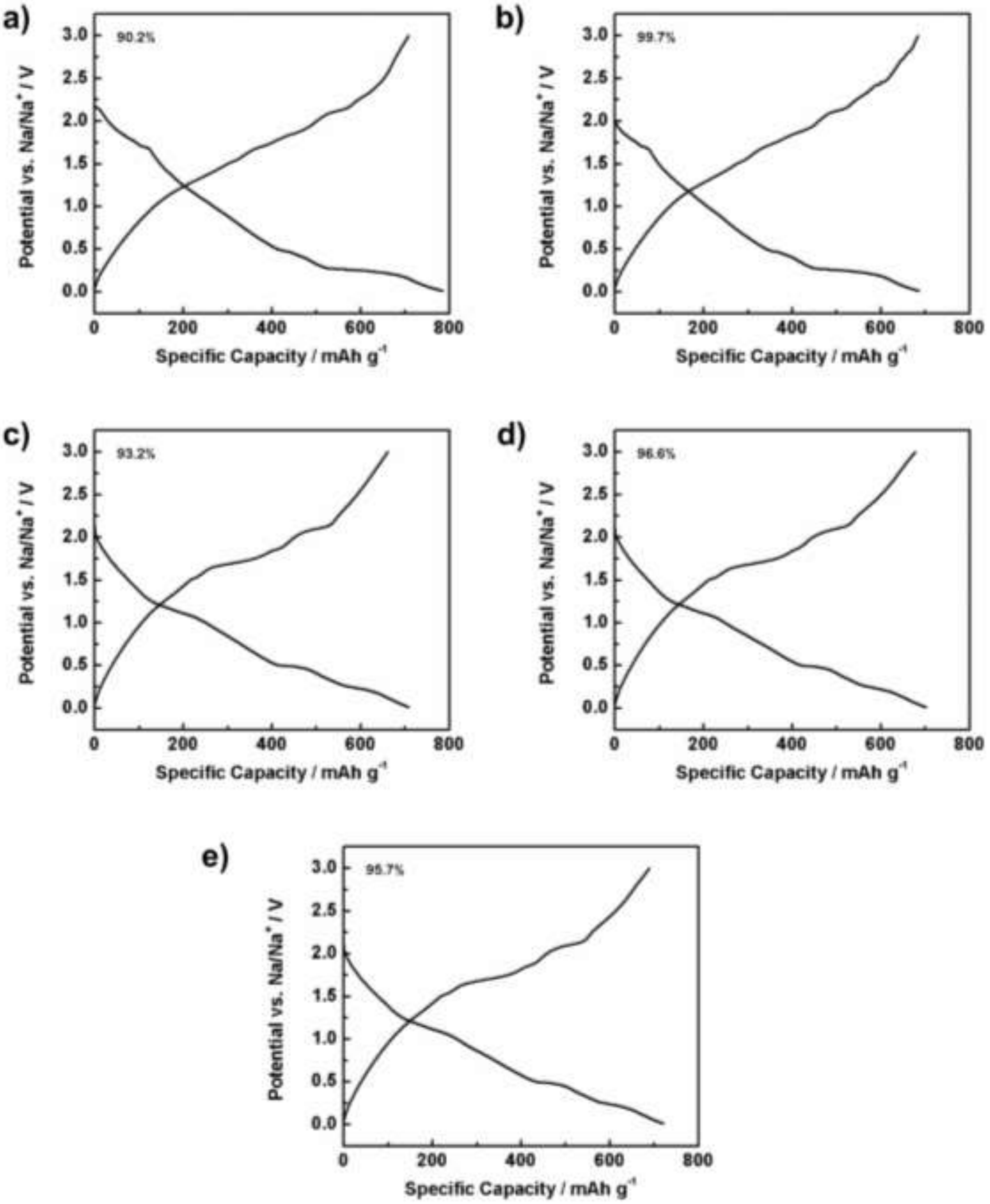

Figure S6. ICE of VS_2/MXene electrodes with five different batteries.

Table S1 The comparison of ICEs and cycling performance among the MXene-based materials for SIBs anodes (NM means not mentioned).

Sample	Cycle number	Current density / A g^{-1}	Capacity retention / %	ICE / %	reference
PDDA-BP/MXene	500	0.1	NM	68.7	[2]
MoS2/MXene	100	0.1	88%	67.4	[3]

MoS2/MXene	70	0.1	96%	62.4	4
MoSe2/MXene	200	1	83%	69.9	5
SnS2/MXene	200	0.1	NM	52.2	6
SnS/MXene	50	0.5	NM	70.4	7
TiO2/MXene	100	0.6	95%	61.1	8
Sb2O3/MXene	100	0.5	NM	59.2	9
Sb/MXene	8000	1	92..%	57	10
NaTi2(PO4)3/MXene	500	0.2	NM	76.3	11
NiCoP/MXene	2000	1	NM	68.7	12
VS2/MXene	500	1	100%	95.08	this work

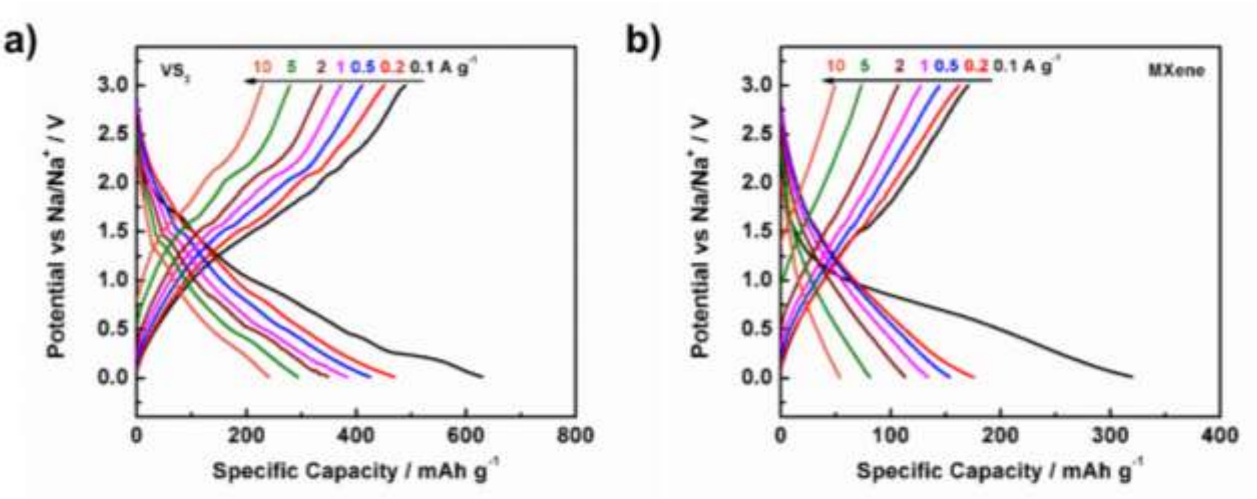

Figure S7. The galvanostatic charge-discharge curves of (a) VS_2 and (b) MXene electrode at various current densities from 0.1 to 10 A g^{-1}.

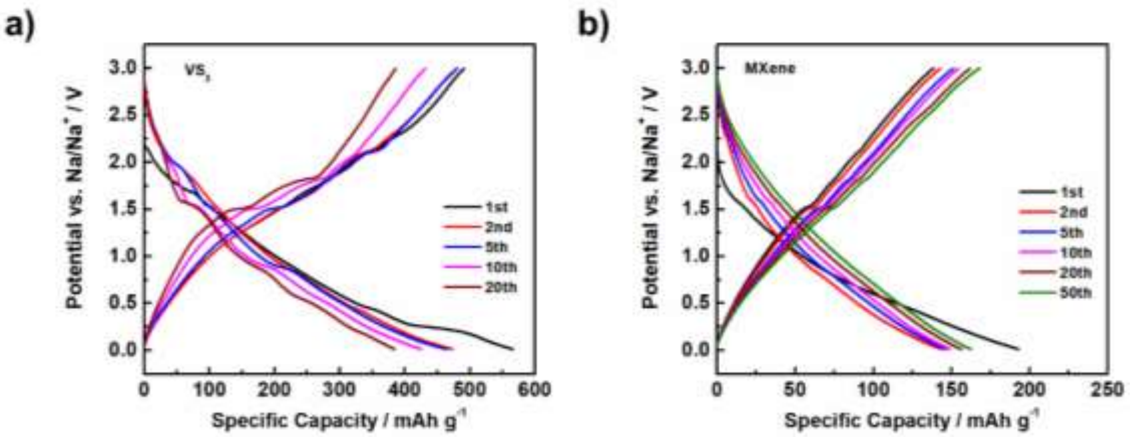

Figure S8. The galvanostatic charge-discharge curves of (a) VS_2 and (b) MXene electrode at a current density of 0.1 A g^{-1}.

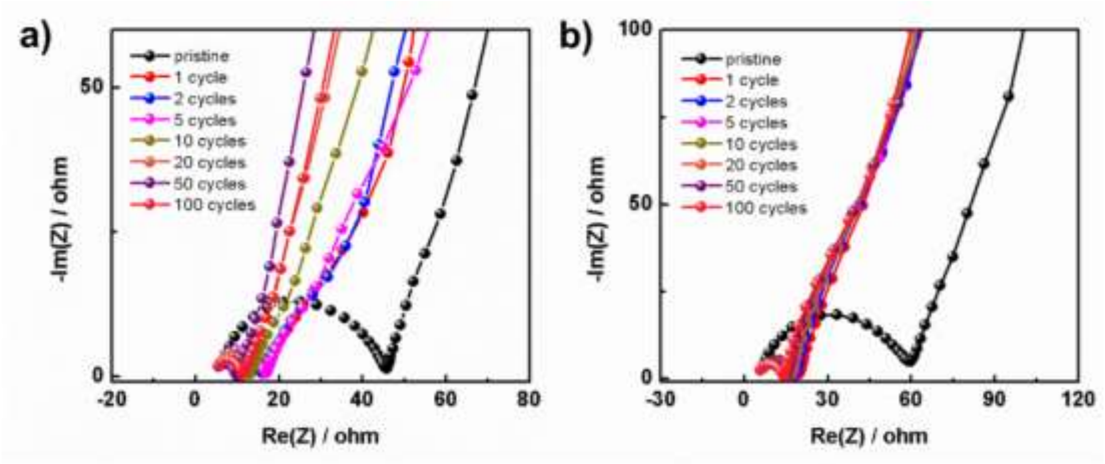

Figure S9. Nyquist plots of the (a) VS_2 and (b) MXene electrode before cycling and after different cycles at full charge states.

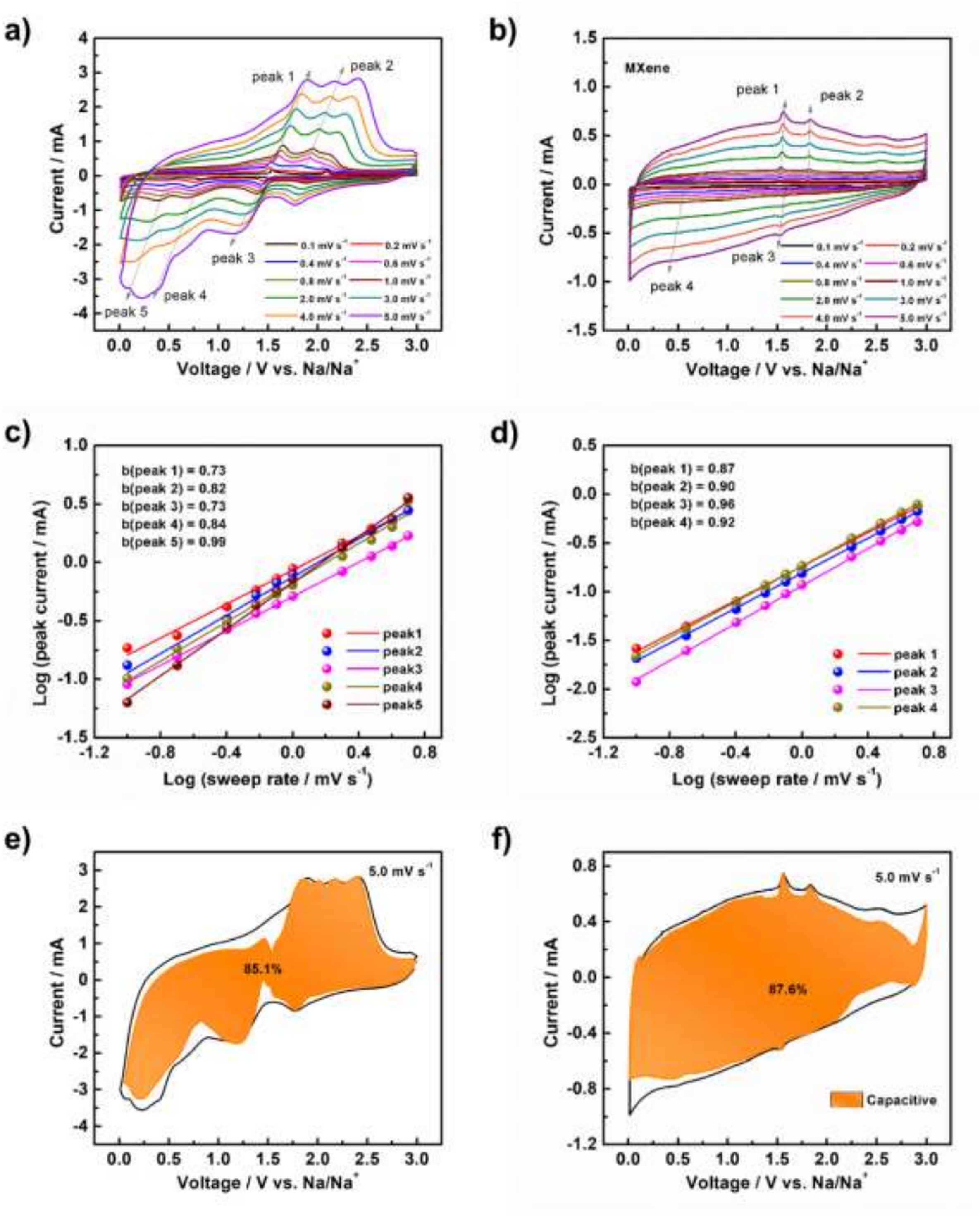

Figure S10. The galvanostatic charge-discharge curves of (a) VS_2 and (b) MXene electrode at a current density of 0.1 A g^{-1}. Relationship between log (peak current) vs. log (sweep rate) for (c) VS_2 and (d) MXene electrode. Capacitive-controlled and diffusion-controlled contributions for (e) VS_2 and (f) MXene electrode at 5.0 mV s^{-1}.

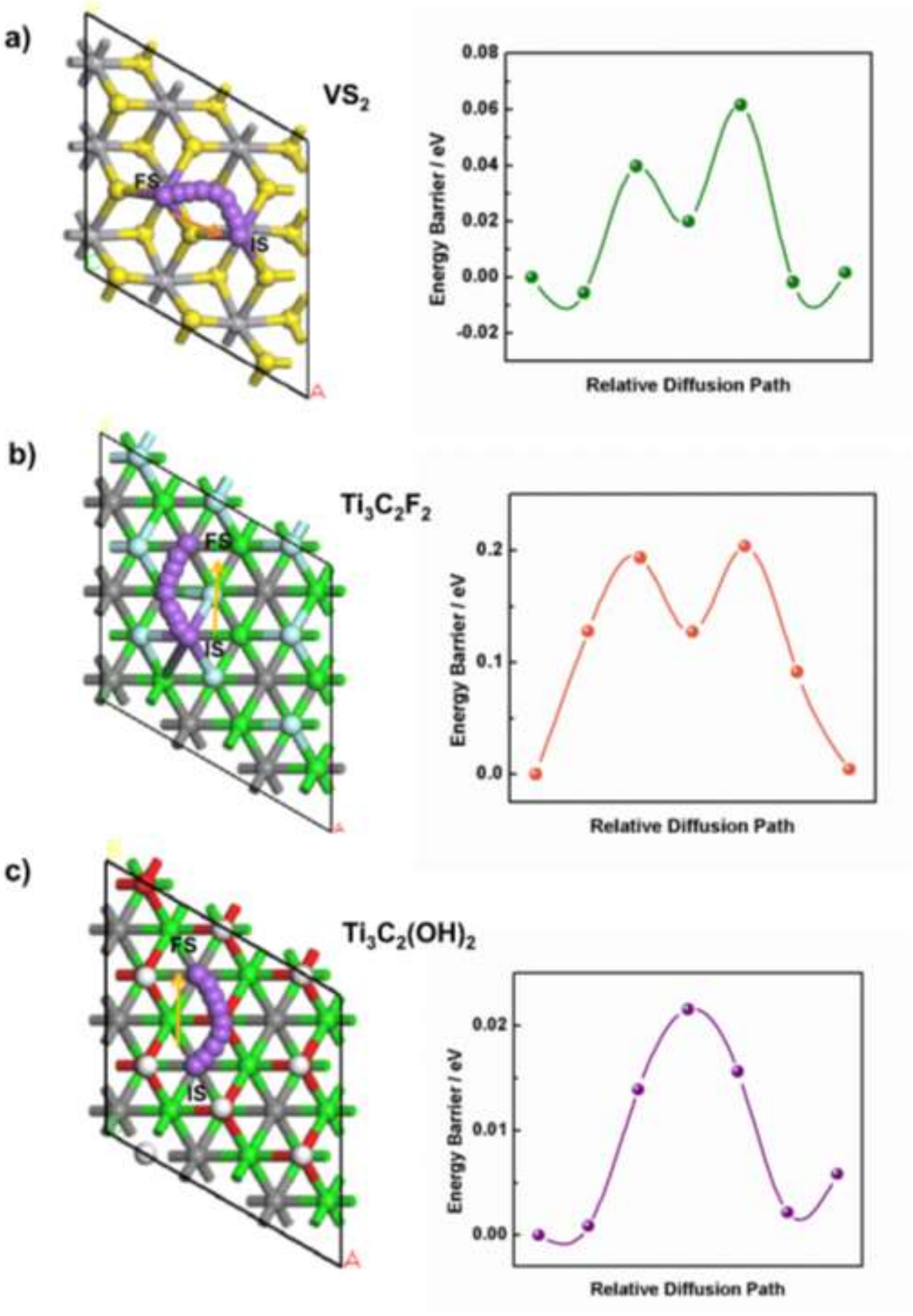

Figure S11. Diffusion path of one Na atom diffusion on the surface of (a) VS_2, (b) $Ti_3C_2F_2$ and (c) $Ti_3C_2(OH)_2$.

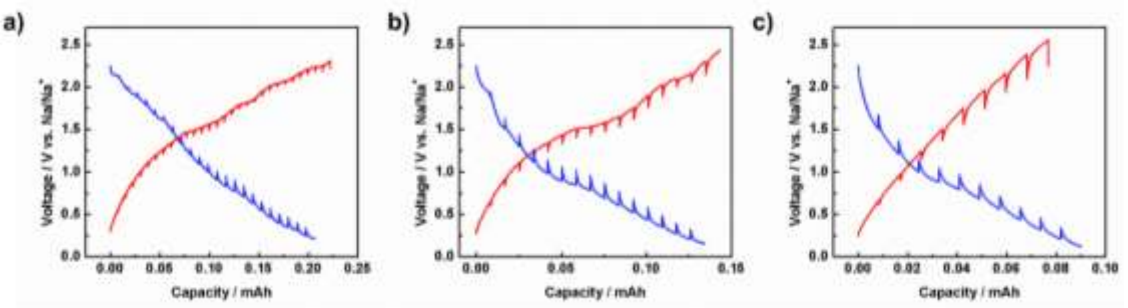

Figure S12. GITT profiles for (a) VS_2/MXene, (b) VS_2 and (c) MXene electrode measured at a current density of 0.1 A g^{-1}.

Table S2. The Bader charges Figure 6f of V, S, Ti, C and F of the $VS_2/Ti_3C_2F_2$ structure.

	V	S_1	S_2	Ti_1	Ti_2	Ti_3	C_1	C_2	F_1	F_2
	2.012151	-1.00075	-1.001427	2.40712	2.314359	2.404202	-2.6987	-2.70126	-0.85806	-0.85899
	2.000791	-0.99996	-1.004207	2.407172	2.31437	2.404187	-2.69876	-2.7012	-0.8581	-0.85901
	2.00713	-1.00352	-1.008985	2.407123	2.314367	2.404201	-2.69865	-2.70124	-0.85808	-0.859
	2.000336	-1.00531	-0.998952	2.407213	2.314368	2.404252	-2.69877	-2.70125	-0.85808	-0.85902
	1.994179	-0.9957	-1.013595	2.407109	2.314371	2.404195	-2.69874	-2.70136	-0.85811	-0.85901
	1.997545	-1.002	-1.001427	2.407223	2.314365	2.404187	-2.69866	-2.7014	-0.85806	-0.859
	1.999412	-1.00717	-1.002485	2.40712	2.314364	2.404199	-2.69873	-2.70123	-0.85805	-0.859
	1.991989	-1.00594	-1.007747	2.407211	2.31436	2.404191	-2.69856	-2.70129	-0.8581	-0.85902
	2.000028	-0.99996	-1.00979	2.407221	2.31437	2.404197	-2.69864	-2.7013	-0.85809	-0.85901
		-0.9957	-1.008985						-0.85808	-0.85901
		-1.00834	-1.001427						-0.85806	-0.85901
		-1.00075	-1.005803						-0.85807	-0.85899
		-1.002	-1.004207						-0.8581	-0.859
		-1.00352	-1.002485						-0.85805	-0.859
		-0.9957	-0.998952						-0.85808	-0.85901
**Sum**	18.00356	-15.0263	-15.0705	21.66451	20.82929	21.63781	-24.2882	-24.3115	-12.8712	-12.8851
**Average**	**2.00**	**-1.00**		**2.38**			**-2.70**		**-0.86**	

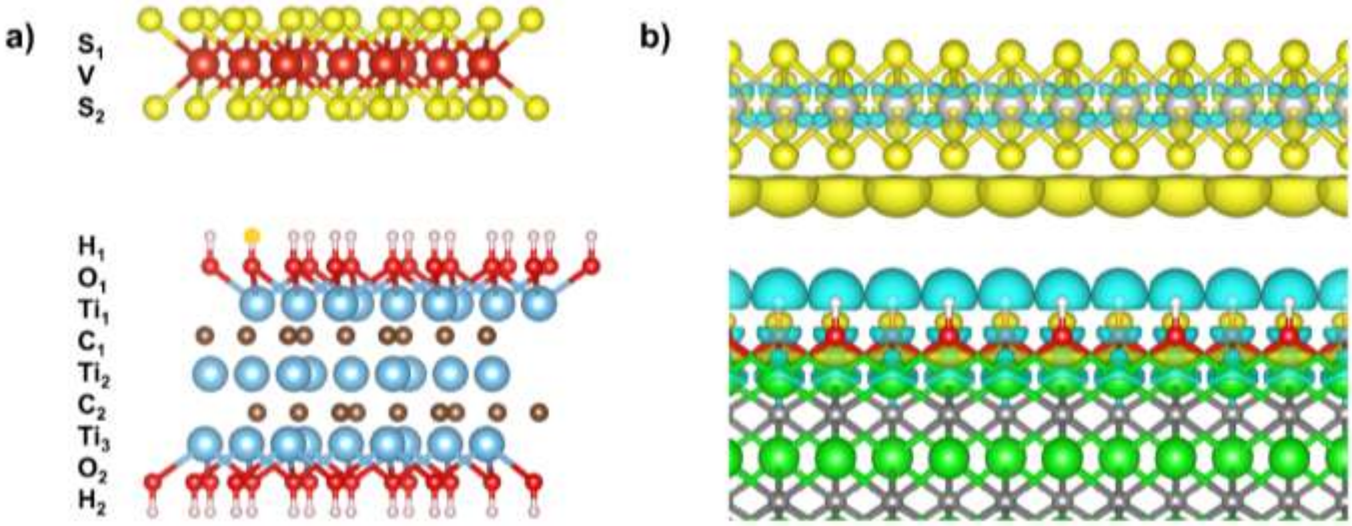

Figure S13. a) Side view and the graphical display of charge differences for $VS_2/Ti_3C_2(OH)_2$ structure.

Table S3. The Bader charges Figure S13a of V, S, Ti, C and O of the $VS_2/Ti_3C_2(OH)_2$ structure.

	V	S_1	S_2	Ti_1	Ti_2	Ti_3	C_1	C_2	O_1	O_2
	1.995372	-1.03242	-1.05476	2.407772	2.324075	2.398258	-2.67764	-2.69119	-1.40105	-1.40217
	1.993935	-1.02931	-1.06224	2.407688	2.323953	2.397544	-2.67755	-2.69103	-1.40016	-1.40313
	2.001153	-1.03231	-1.06148	2.40738	2.323979	2.39714	-2.67771	-2.69097	-1.40288	-1.40185
	1.994157	-1.02671	-1.0548	2.40738	2.324319	2.397917	-2.67717	-2.6914	-1.40007	-1.40228
	1.99541	-1.03025	-1.05476	2.407353	2.323942	2.398079	-2.67732	-2.69132	-1.40105	-1.40377
	1.999992	-1.03586	-1.06285	2.407705	2.324218	2.397589	-2.67751	-2.69123	-1.40019	-1.40288
	2.006371	-1.033	-1.06358	2.398403	2.324071	2.398403	-2.67705	-2.69143	-1.40074	-1.40204
	2.002153	-1.03231	-1.05713	2.407567	2.32386	2.397907	-2.67709	-2.6912	-1.39975	-1.40185
	2.006648	-1.03732	-1.0548	2.407498	2.323866	2.397017	-2.67733	-2.69101	-1.40007	-1.40286
		-1.04073	-1.06887						-1.39959	-1.40233
		-1.02671	-1.06533						-1.40021	-1.40228
		-1.033	-1.05476						-1.40105	-1.40204
		-1.03242	-1.05713						-1.39975	-1.40217
		-1.02931	-1.06224						-1.40016	-1.40313
		-1.02671	-1.06148						-1.39784	-1.40228
**Sum**	17.99519	-15.47837	-15.89623	21.65875	20.91628	21.57985	-24.096358	-24.220772	-21.004581	-21.037057
**Aver-age**	**2.00**	**-1.05**		**2.38**			**-2.68**		**-1.40**	

References

[1] Sun, R.; Wei, Q.; Sheng, J.; Shi, C.; An, Q.; Liu, S.; Mai, L. Novel layer-by-layer stacked VS_2 nanosheets with intercalation pseudocapacitance for high-rate sodium ion charge storage. *Nano Energy*, **2017**, 35, 396-404.

[2] Zhao, R.; Qian, Z.; Liu, Z.; Zhao, D.; Hui, X.; Jiang, G.; Wang, C.; Yin, L. Molecular-level heterostructures assembled from layered black phosphorene and Ti_3C_2 MXene as superior anodes for high-performance sodium ion batteries. *Nano Energy*, **2019**, 65, 104037.

[3] Wu, Y.; Nie, P.; Jiang, J.; Ding, B.; Dou, H.; Zhang, X. MoS_2-nanosheet-decorated 2D titanium carbide (MXene) as high-performance anodes for sodium-ion batteries. *Chem. Electro. Chem.*, **2017**, 4, 1560-1565.

[4] Du, G.; Tao, M.; Gao, W.; Zhang, Y.; Zhan, R.; Bao, S.; Xu, M. Preparation of $MoS_2/Ti_3C_2T_x$ composite as anode material with enhanced sodium/lithium storage performance. *Inorg. Chem. Front.*, **2019**, 6, 117-125.

[5] Xu, E.; Zhang, Y.; Wang, H.; Zhu, Z.; Quan, J.; Chang, Y.; Li, P.; Yu, D.; Jiang, Y. Ultrafast kinetics net electrode assembled via $MoSe_2$/MXene heterojunction for high-performance sodium-ion batteries. *Chem. Eng. J.*, **2020**, 385, 123839.

[6] Wu, Y.; Nie, P.; Wu, L.; Dou, H.; Zhang, X. 2D MXene/SnS_2 composites as high-performance anodes for sodium ion batteries. *Chem. Eng. J.*, **2018**, 334, 932-938.

[7] Zhang, Y.; Guo, B.; Hu, L.; Xu, Q.; Li, Y.; Liu, D.; Xu, M. Synthesis of SnS nanoparticle-modified MXene ($Ti_3C_2T_x$) composites for enhanced sodium storage. *J. Alloy. Compd.*, **2018**, 732, 448-453.

[8] Wang, P.; Lu, X.; Boyjoo, Y.; Wei, X.; Zhang, Y.; Guo, D.; Sun, S.; Liu, J. Pillar-free TiO_2/Ti_3C_2 composite with expanded interlayer spacing for high-capacity sodium ion batteries. *J. Power Sources*, **2020**, 451, 227756.

[9] Guo, X.; Xie, X.; Choi, S.; Zhao, Y.; Liu, H.; Wang, C.; Chang, S.; Wang, G. Sb_2O_3/MXene($Ti_3C_2T_x$) hybrid anode materials with enhanced performance for sodium-ion batteries. *J. Mater. Chem. A*, **2017**, 5, 12445-12452.

[10] Chen, H.; Chen, N.; Zhang, M.; Li, M.; Gao, Y.; Wang, C.; Chen, G.; Du, F. $Ti_3C_2T_x$ MXene decorated with Sb nanoparticles as anodes material for sodium-ion batteries. *Nanotechnology*, **2019**, 30, 134001.

[11] Yang, C.; Sun, X.; Zhang, Y. R.; Liu, Y.; Zhang, Q. A.; Yuan, C. Z. Facile synthesis of hierarchical $NaTi_2(PO_4)_3/Ti_3C_2$ nanocomposites with superior sodium storage performance. *Mater. Lett.*, **2019**, 236, 408-411.

[12] Zhao, D.; Zhao, R.; Dong, S.; Miao, X.; Zhang, Z.; Wang, C.; Yin, L. Alkali-induced 3D crinkled porous Ti_3C_2 MXene architectures coupled with NiCoP bimetallic phosphide nanoparticles as anodes for high-performance sodium-ion batteries. *Energy Environ. Sci.*, **2019**, 12, 2422-2432.
